# Supplementary material for: Improved Media Formulations for Primary Cell Cultures Derived from a Colonial Urochordate
Source: Cells. 2023 Jun 23;12(13):1709. doi: 10.3390/cells12131709 (PMC10340598; doi:10.3390/cells12131709)
Supplement: Supplementary file 1 [file cells-12-01709-s001.zip › cells-2452223-supplementary.pdf]

Improved media formulations for primary cell cultures derived from a colonial urochordate

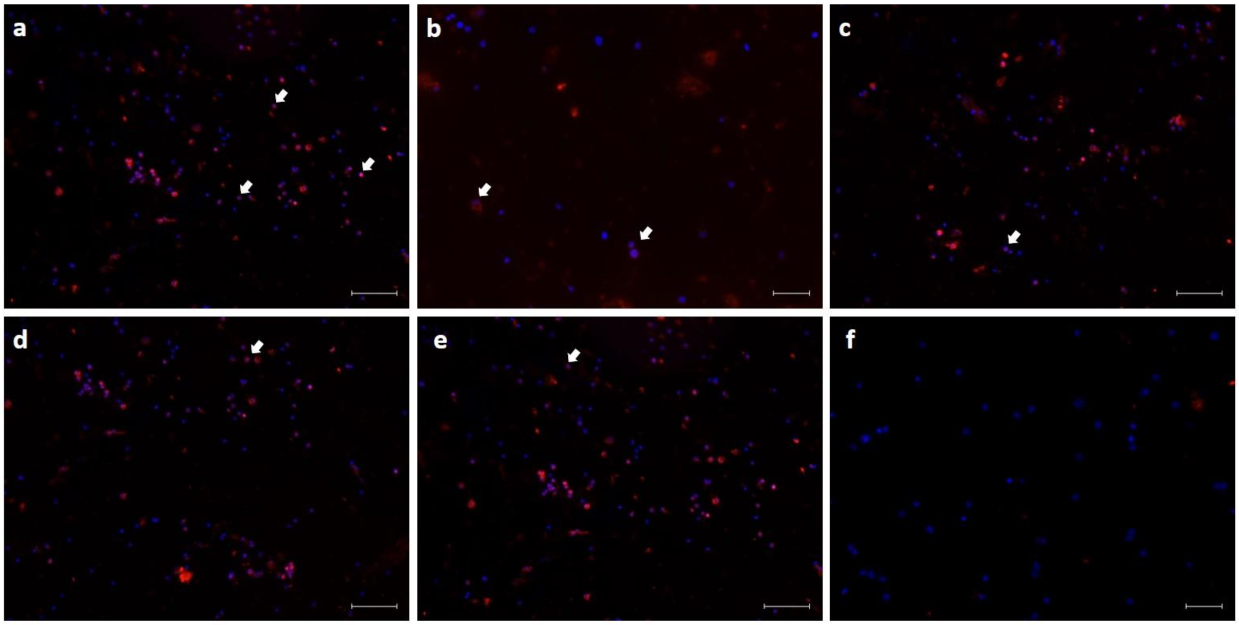

Figure S1. Immunofluorescence staining of *B. schlosseri* primary cultures under five media conditions at onset. a: TGM1. b: TGM2. c: TGM3. d: TGM4. e: TGM5. Blue dots are DAPI stained cell nuclei, red dots indicate $PCNA^+$ stained nuclei and pink cells (some marked with arrows) indicating $PCNA^+$ cells. f: negative control depict DAPI stained cell nuclei and unspecific staining of PCNA antibody. Scale bars: 20 µm in b and f, 50 µm in a, c, d and e.

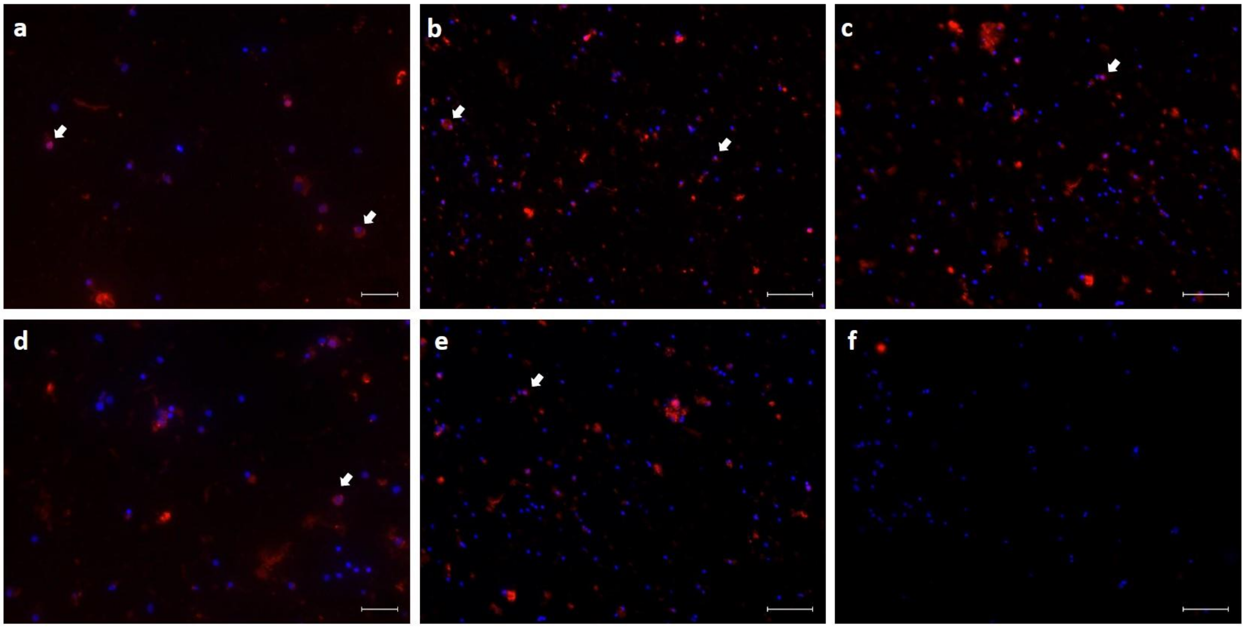

Figure S2. Immunofluorescence staining of *B. schlosseri* primary cultures under five media conditions at 24 h from initiation. a: TGM1. b: TGM2. c: TGM3. d: TGM4. e: TGM5. Blue dots are DAPI stained cell nuclei, red dots indicate $PCNA^+$ stained nuclei and pink cells (some marked with arrows) indicating $PCNA^+$ cells. f: negative control depict

DAPI stained cell nuclei and unspecific staining of PCNA antibody. Scale bars: 20 µm in a and d, 50 µm in b, c, e and f.

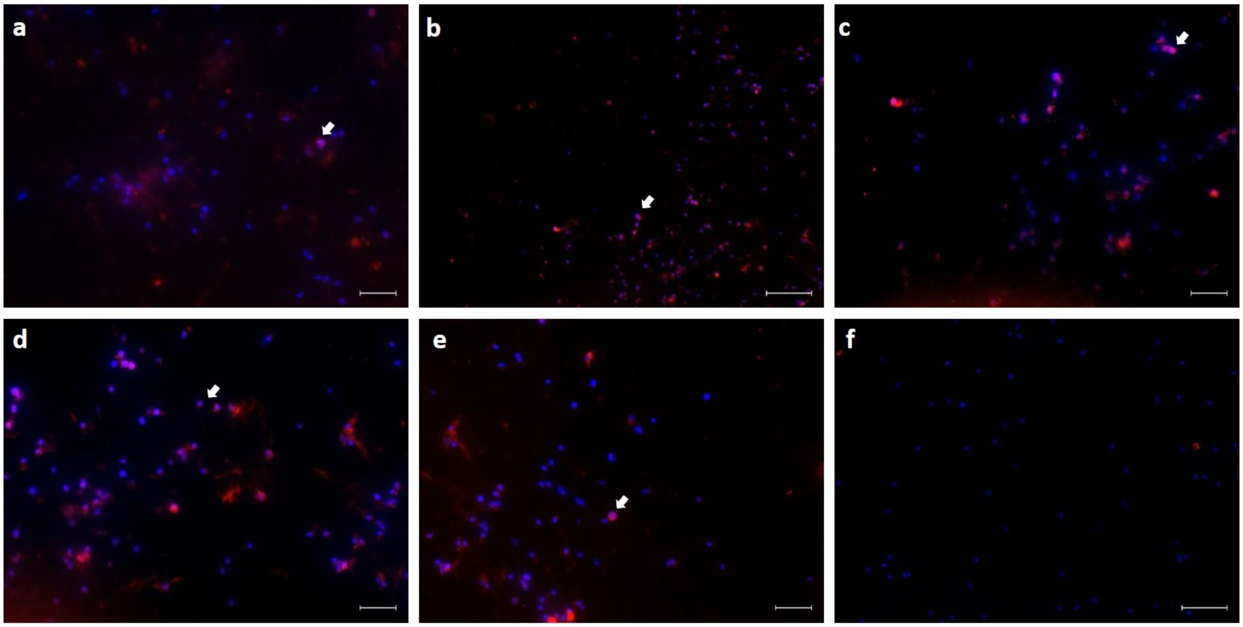

Figure S3. Immunofluorescence staining of *B. schlosseri* primary cultures under five media conditions at 3 days from initiation. a: TGM1. b: TGM2. c: TGM3. d: TGM4. e: TGM5. Blue dots are DAPI stained cell nuclei, red dots indicate PCNA⁺ stained nuclei and pink cells (some marked with arrows) indicating PCNA⁺ cells. f: negative control depict DAPI stained cell nuclei and unspecific staining of PCNA antibody. Scale bars: 20 µm in a, c, d and e, 50 µm in b and f.

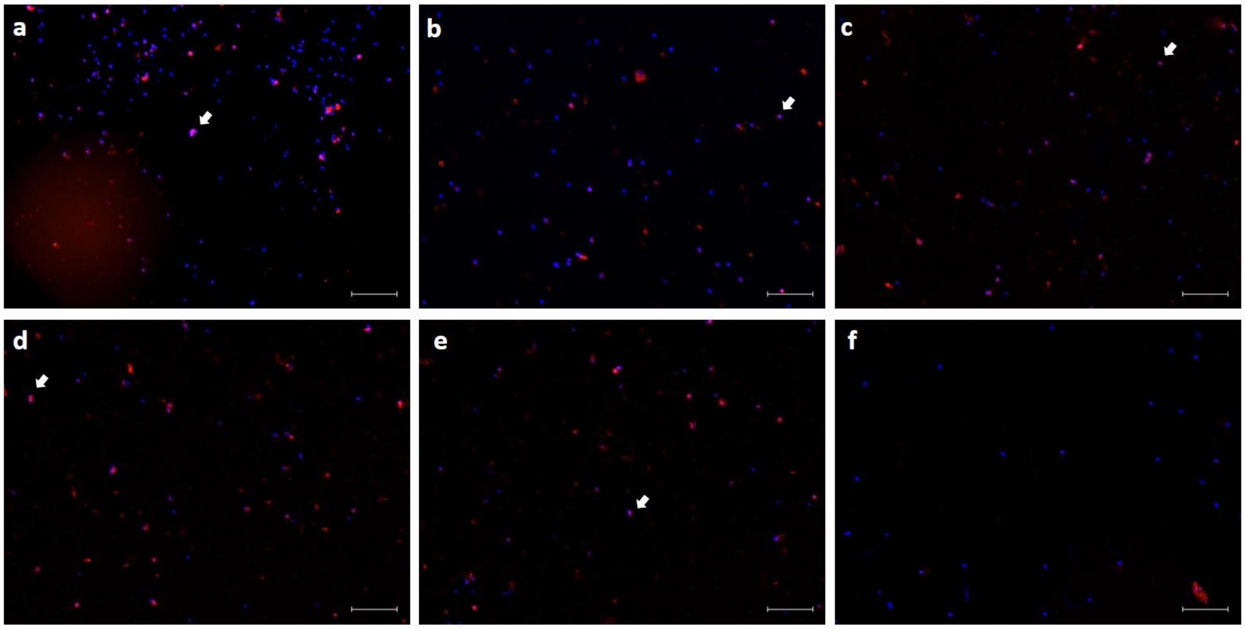

Figure S4. Immunofluorescence staining of *B. schlosseri* primary cultures under five media conditions at 8 days from initiation. a: TGM1. b: TGM2. c: TGM3. d: TGM4. e: TGM5. Blue dots are DAPI stained cell nuclei, red dots

indicate PCNA⁺ stained nuclei and pink cells (some marked with arrows) indicating PCNA⁺ cells. f: negative control depict DAPI stained cell nuclei and unspecific staining of PCNA antibody. Scale bars = 50 μm.

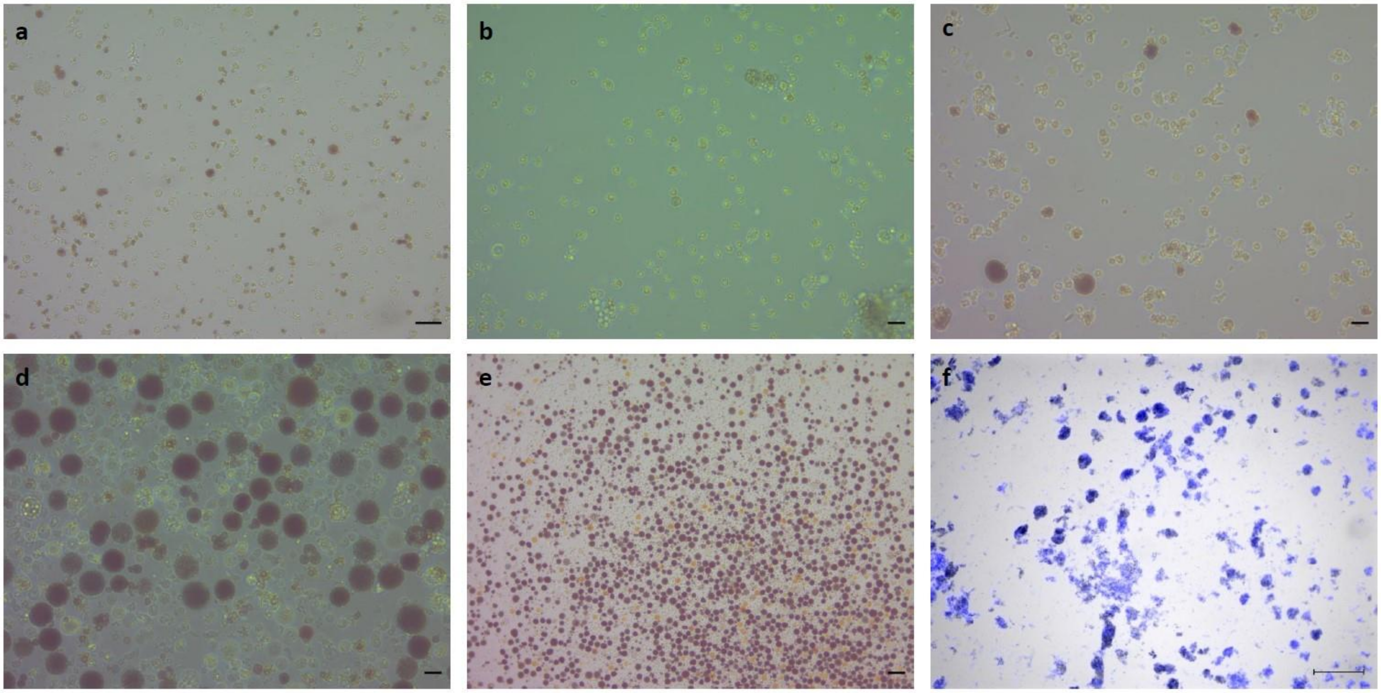

Figure S5. *B. schlosseri* primary blood cell cultures in medium TGM1: at onset (a), 24 h (b), days 3 (c), 8 (d) and 15 (e). f: fungi contamination at day 14 detected by calcofluor white staining. Scale bars: 10 μm in b, c and d, 20 μm in a, 80 μm in e, 100 μm in f.

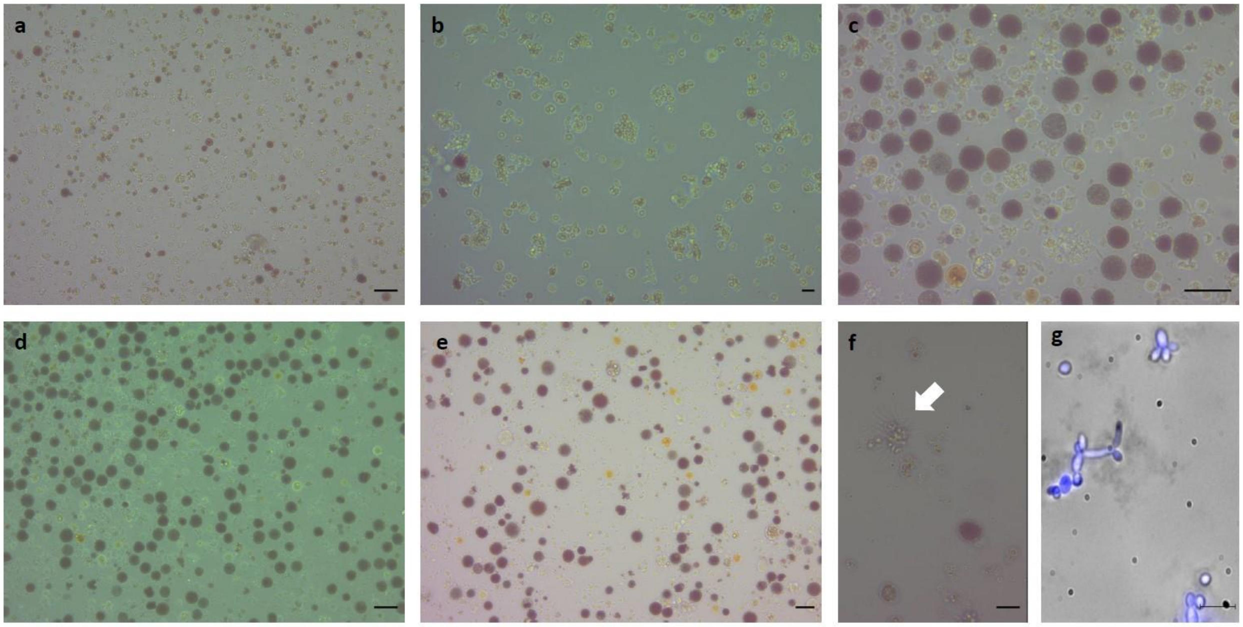

Figure S6. *B. schlosseri* primary blood cell cultures in medium TGM2: at onset (a), 24 h (b), days 3 (c), 8 (d) and 12 (e). Thraustochytrids contamination (arrow) at day 10 (f). g: yeast contamination at day 14 detected by calcofluor white staining. Scale bars: 10 μm in b, f and g, 20 μm in a and e, 40 μm in c and d.

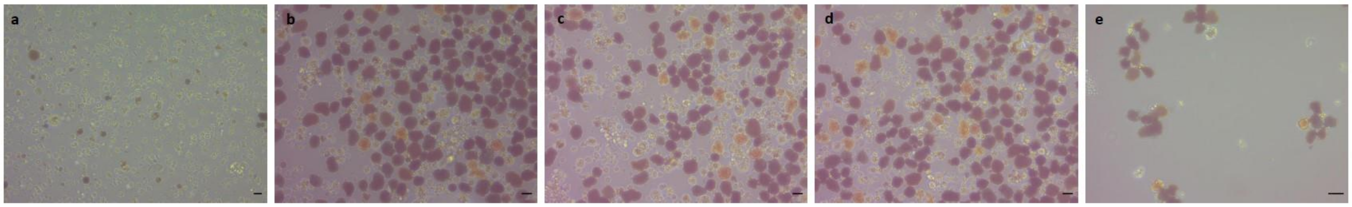

Figure S7. *B. schlosseri* primary blood cell cultures in medium TGM3: at onset (a), 24 h (b), days 3 (c), 8 (d) and 10 (e). Scale bars: 10 μm.

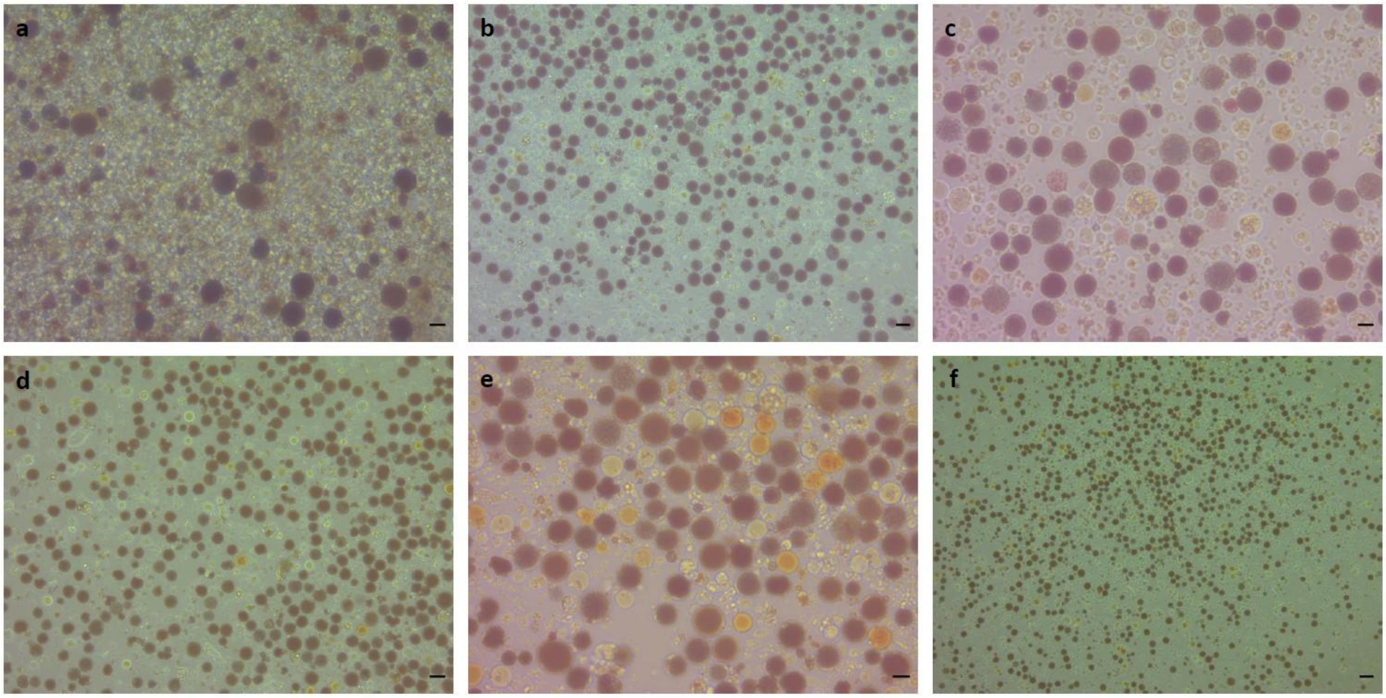

Figure S8. *B. schlosseri* primary blood cell cultures in medium TGM4: at onset (a), 24 h (b), days 3 (c), 8 (d), 17 (e) and 25 (f). Scale bars: 10 μm in b-d, 20 μm in a, e and f.

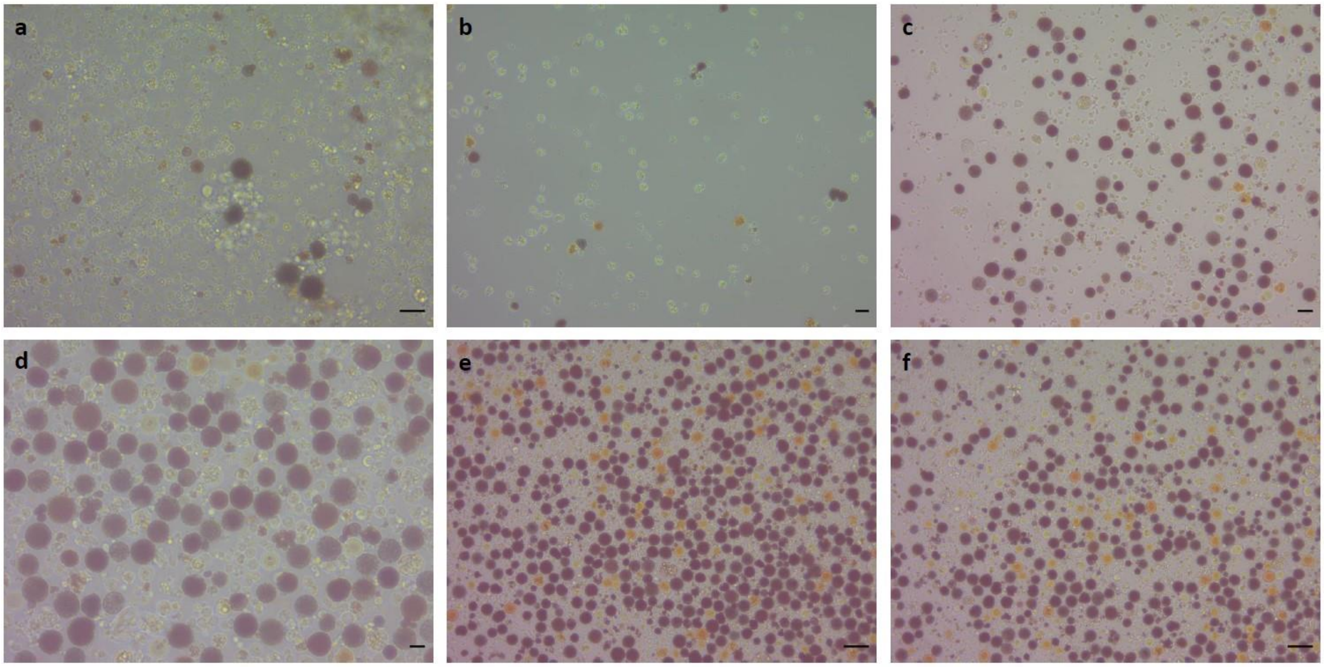

Figure S9. *B. schlosseri* primary blood cell cultures in medium TGM5: at onset (a), 24 h (b), days 3 (c), 8 (d), 17 (e) and 23 (f). Scale bars: 10 μm in a-f, 20 μm in f.

Table S1. Comparisons of media TGM1, TGM2, TGM3, TGM4 and TGM5 compositions. L-Glu - L-Glutamine, Hep - HEPES buffer, PSA - Penicillin Streptomycin Amphotericin b, Genta – Gentamicin, PS - Penicillin Streptomycin, SP - Sodium Pyruvate, FBS - Fetal Bovine Serum, DMEM - DMEM/F-12[HAM] 1:1 basal medium, RPMI - RPMI basal medium 1640, ASW – artificial sea water. +/- indicates the presence or absence of components in each medium.

Medium/Component	L-Glu	Hep	PSA	Genta	PS	SP	FBS	DMEM	RPMI	ASW
TGM1	+	+	-	-	+	+	+	+	-	-
TGM2	+	+	-	-	+	+	+	-	+	-
TGM3	+	+	+	+	-	+	+	-	-	+
TGM4	+	+	-	+	+	+	+	+	-	+
TGM5	+	+	-	-	+	+	+	+	-	+
